# Is it time for China to prioritize pan-genotypic regimens for treating patients with hepatitis C?

**DOI:** 10.1186/s12962-024-00519-2

**Published:** 2024-02-06

**Authors:** Yusi Tu, Xiangyan Tang, Dachuang Zhou, Hanqiao Shao, Leyi Liang, Wenxi Tang

**Affiliations:** 1 Center for Pharmacoeconomics and Outcomes Research, China Pharmaceutical University, Nanjing, 211198 China; 2 Department of Public Affairs Management, School of Lnternational Pharmaceutical Business, China Pharmaceutical University, 639#Longmian Road, Nanjing, 211198 China

**Keywords:** Hepatitis C, Pan-genotypic regimens, Cost-effectiveness analysis, Decision-analytic Markov model

## Abstract

**Introduction:**

The treatment of hepatitis C has entered the pan-genotypic era, but the effectiveness is not good for the genotype 3b patients who have a large proportion in China. The guidelines for hepatitis C recommend the use of gene-specific regimens when the regional 3b prevalence rate greater than 5%. This study is to explore rationality of this proportion and the cost-effectiveness to implement pan-genotypic regimens in China.

**Methods:**

A decision Markov model was developed from the health system perspective to evaluate the effectiveness and cost-effectiveness between pan-genotypic and gene-specific treatment regimens for hepatitis C patients. Additionally, we set a regional genotype 3b patient proportion of 0–100% to explore at which proportion it is necessary to perform genotype identification and typing therapy on patients. Model parameters were derived from published literature and public databases. Effectiveness was measured by cured patient numbers, newly diagnosed cases of decompensated cirrhosis, hepatocellular carcinoma, need for liver transplantation, and quality-adjusted life years (QALYs). Cost-effectiveness outcomes included costs and the incremental cost-effectiveness ratio (ICER). The 1–3 times 2022 Chinese per capita gross domestic product was used as the willingness-to-pay threshold. One-way and probabilistic sensitivity analyses were performed to assess the uncertainty of the model parameters.

**Results:**

Compared with gene-specific regimens, pan-genotypic regimens resulted in an additional 0.13 QALYs and an incremental cost of $165, the ICER was $1,268/QALY. From the view of efficacy, the pan-genotypic regimens cured 5,868 more people per 100,000 patients than gene-specific regimens, avoiding 86.5% of DC cases, 64.6% of HCC cases, and 78.2% of liver transplant needs. Identifying 3b patients before treatment was definitely cost-effectiveness when their prevalence was 12% or higher. The results remained robust in sensitivity analyses.

**Conclusions:**

In China, the prioritized recommendation of pan-genotypic therapeutics proves to be both cost-effective and efficacious. But, in regions where the prevalence of genotype 3b exceeds 12%, it is necessary to identify them to provision of more suitable therapies.

**Supplementary Information:**

The online version contains supplementary material available at 10.1186/s12962-024-00519-2.

## Introduction

China has the largest number of people with hepatitis C infection in the world [1]. In China, the number of hepatitis C virus infections reached nearly 10 million by 2020 [2]. According to the China Health Statistics Yearbook, the reported incidence rate of hepatitis C in China increased from 9.93/10,000 in 2009 to 16.02/10,000 in 2019 [3]. Patients with untreated chronic hepatitis C infection may develop serious liver-related complications, such as decompensated cirrhosis (DC) and hepatocellular carcinoma (HCC), resulting in significant clinical and economic burden.

The introduction of direct-acting antiviral agents (DAAs) in 2010 marked a transformative moment, rendering hepatitis C a curable condition. Two principal categories of DAAs have surfaced: gene-specific agents and pan-genotypic agents. Gene-specific agents necessitate genotyping tests, a procedure demanding specialized expertise and expensive equipment, making it challenging to implement in low- and middle-income countries [4]. The advent of pan-genotypic agents streamlined the treatment process by obviating the necessity for genotypic tests while retaining the benefits of traditional DAAs [5]. Nevertheless, pan-genotypic regimens are relatively expensive and exhibit reduced efficacy in patients with hepatitis C genotype 3b [6, 7]. In practice, selecting pan-genotypic regimens, despite the disadvantages, enhances overall treatment accessibility and can extend benefits to a broader patient population because of interruption of transmission. Conversely, opting for gene-specific treatment is cheaper, but may inadvertently restrict treatment access for patients in resource-constrained regions [8, 9].

The World Health Organization has endorsed pan-genotypic regimens as the preferred treatment for adult hepatitis C patients, and several nations, including the United States and France, have adhered to this recommendation. In contrast, countries such as Japan, Canada, and China, persist in their prioritization of gene-specific regimens due to price and efficacy concerns. Currently, the price of pan-genotypic regimens significantly decreased following their inclusion in China's health insurance reimbursement system in 2020. Effective second-line therapies such as sofosbuvir/velpatasvir/voxilaprevir (SOF/VEL/VOX), have been developed for patients who did not respond to initial DAAs, achieving a cure rate of approximately 90% [10]. The primary focus is to explore whether China should consider changing its preferred treatment regimen, given the current pricing of pan-genotypic DAAs and the prevalence of Hepatitis C.

Furthermore, hepatitis C is transmitted through various routes. While nosocomial transmission is now effectively controlled, the challenges persist in controlling transmission among individuals engaged in drug use and those involved in high-risk sexual behaviors [11, 12]. The majority of this population is infected with subtypes 3 and 6 [13–15], signifying that patients with genotype 3b will account for an increased proportion of patients with hepatitis C in the future [16]. Using pan-genotypic regimens may not be the best choice for 3b patients, but the process of identifying these patients imposes time and testing costs on non-3b patients. The Chinese Guideline for the prevention and treatment of hepatitis C (2022 version) recommend the testing for genotype is required even use pan-genotypic regimens when the regional prevalence of genotype 3b more than 5% [2]. Based on this, the second question in this study is whether it is necessary to identify 3b patients and change them to more effective regimens in regions that promote pan-genotypic regimens.

In the present study, our objective was to compare the cost-effectiveness and effectiveness of using pan-genotypic regimens with gene-specific regimens from the Chinese health system perspective.  Additionally, we aimed to explore the necessity of identifying genotype 3b patients and switching regimens in regions prioritizing pan-genotypic regimens.

## Methods

### Study overview

From the perspective of the Chinese health system, we developed a decision-analytic Markov model based on established models that have been widely utilized in hepatitis C research [17–19]. The model was used to simulate the progression of hepatitis C disease under various treatment pathways. The model was built by Microsoft Excel 2016 and updated with recent Chinese-specific data. This study was performed in 2023 and followed the Consolidated Health Economic Evaluation Reporting Standards (CHEERS) reporting guideline [20]. All data were publicly available from published studies and reports that exempted this study from requiring approval from an institutional review board.

This study aimed to answer two questions: (1) "Should pan-genotypic regimens be prioritized in China?". To answer this question, we compared the differences in efficacy and cost-effectiveness between the use of pan-genotypic regimens and gene-specific regimens for adult Chinese patients with hepatitis C. We also considered the impact of simulation years and the price of pan-genotypic regimens on the results when answering this question. (2) "Is it necessary to identify genotype 3b patient in the regions where promoting pan-genotypic regimens". We compared the effectiveness and cost-effectiveness of two approaches: in areas where the use of pan-genotypic regimens are promoted, genotyping the entire patient population to identify subtype 3b patients and changing their regimen to a more effective one, versus not conducting genotyping tests. We established 100 districts with 3b patient proportion varying from 0 to 100% and varied the proportion incrementally by one percent each time. For the sake of simplicity in our comparison, we categorized the population into two groups: 3b patients and non-3b patients. Except for the 3b patients in the group requiring genotyping tests, who received sofosbuvir/velpatasvir (SOF/VEL) plus ribavirin (RBV), all other patients were treated with SOF/VEL.

### Model description

**Fig. 1:**
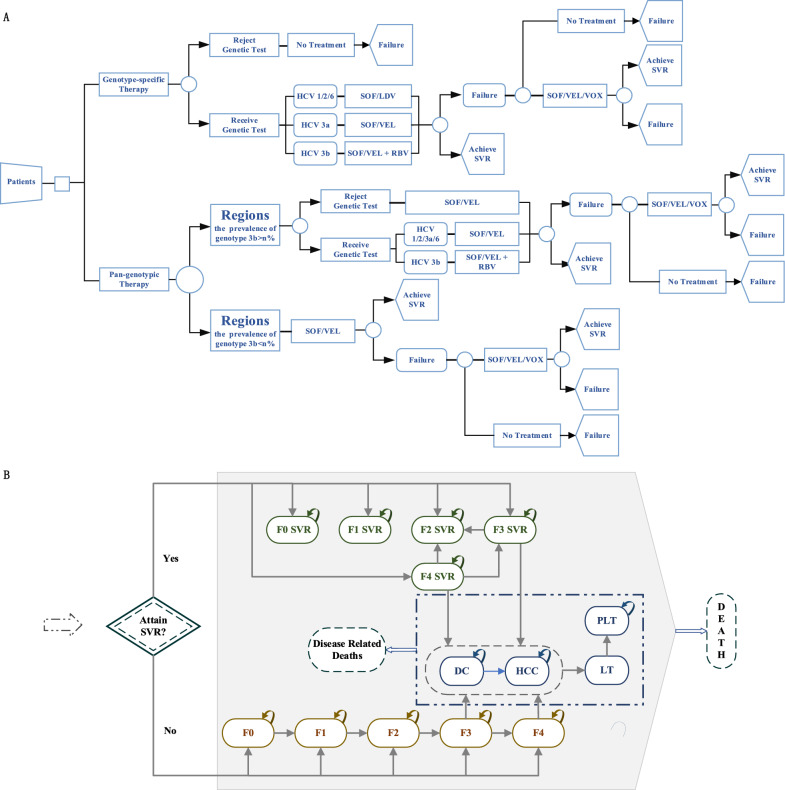
Decision analysis model structure. **A** decision tree model structure; **B** markov model structure. *SVR* sustained virologic response, *F0–F4* METAVIR fibrosis score, *DC* decompensated cirrhosis, *HCC* hepatocellular carcinoma, *LT* liver transplantation(first year), *PLT* post-liver transplantation (> 1 year), *SOF* Sofosbuvir, *VEL* Velpatasvir, *LDV* Ledipasivr, *VOX* Voxilaprevir, *RBV* ribavirin

This model consisted of a decision tree model and a 16-state Markov model and model structure is shown in Fig. 1.

### Decision tree model structure

A decision tree model was built to simulate the outcomes of patients with hepatitis C receiving different treatment options.

From January 2020, sofosbuvir/ledipasvir (SOF/LDV), SOF/VEL, and grazoprevir/elbasvir(GZR/EBR) accounted for almost the entire DAAs market in China [21]. Therefore, we chose SOF/VEL to represent the pan-genotype regimens. Because GZR/EBR can only treat type 1 patients and shares similar treatment duration, efficacy, and cost attributes with SOF/LDV, we chose SOF/LDV to represent gene-specific DAAs. Determine treatment regimens for different patient cohorts based on the advice in guidelines [2]. For the gene-specific regimen, treatment selection would be guided by genotyping test results. Patients with genotypes 1, 2, and 6 received SOF/LDV, genotype 3a patients were treated with SOF/VEL, and genotype 3b patients were treated with SOF/VEL plus RBV. For the pan-genotypic regimen, in regions where the prevalence of 3b is less than 5%, genotyping was unnecessary, and all patients received SOF/VEL. In areas where the 3b prevalence exceeded 5%, genotyping was needed to identify 3b patients. These 3b patients were treated with SOF/VEL plus RBV, while other patients were treated with SOF/VEL. Therefore, we would follow these rules to differentiate between patients choosing pan-genotypic treatment in question 1 to make it more realistic. All medication courses were 12 weeks. Sustained virologic response (SVR) rates for each treatment and the incidence of serious adverse reactions were estimated from real-world research [22–24] and clinical trials (Additional file 1: Table S1) [6, 7, 25].

Key assumptions in the decision tree model structure were that, first, for patients who must choose treatment based on the genotyping test results, we hypothesized that 5.8% of patients would be lost due to the absence of genotyping facility or other reasons [8, 26, 27]. Second, 75% of patients who failed initial treatment were assumed to continue treatment with the SOF/VEL/VOX regimen, while those who failed second-line treatment were no longer receiving antiviral therapy [28–30]. Third, due to the short treatment time and efficacy of direct antiviral therapy, which needed only oral medication, we assumed 100% compliance with treatment and patients would not reinfect and relapse after obtaining SVR.

### Markov model structure

Patients would transition to the Markov model based on their liver fibrosis stage and the treatment outcomes from the decision tree model. Patients who did not achieve SVR entered the natural history model, while those who were successfully cured entered the SVR model. The natural Markov model was constructed based on the natural history of hepatitis C [31, 32], including five liver fibrosis states distinguished by the METAVIR fibrosis scores(F0, no fibrosis; F1, portal fibrosis without septa; F2, portal fibrosis with few septa; F3, numerous septa without fibrosis; and F4, cirrhosis), the decompensated cirrhosis state, the hepatocellular carcinoma state, the liver transplant state(first year), the post liver transplant state (> 1 year), the liver-related disease death state and the natural death state. The difference between the SVR model and the natural history model was that in the SVR model, patients in stages F0-F2 would not continue to progress in their disease state after obtaining SVR, whereas patients in stages F3 and F4 would continue progressing at a lower probability than those who do not achieve SVR [33]. Progression rates between different disease states were estimated from published meta-analyses [34], observational studies [35, 36], and other cost-effectiveness studies [19, 37] (Additional file 1: Table S2). The model was run with a 1-year cycle and run for 30 years.

### Cohort characteristics

The population cohort in the decision-analytic Markov model represented hepatitis C patients in China. Over the past five years, the average number of newly confirmed cases of hepatitis C in China has been approximately 210,000 per year [38]. According to retrosepctive study, 28,102 patients were treated with DAAs in 2020, and 49,592 in 2021 [21](23% of newly diagnosed cases). We assumed that 50% of patients would be treated with DAAs in 2022. The simulated cohort size was set at 100,000. The hepatitis C virus genotype distribution, liver disease status distribution, and age distribution were obtained from published studies (Additional file 1: Table S3) [39–41]. Considering the average age of the hepatitis C patients receiving treatment [41], the appropriate age for liver transplantation [42], and the average life expectancy in China [43], the model was designed to run for a maximum of 30 cycles. Age-specific annual mortality rates for the general population were from the National Bureau of Statistics.

### Cost and utility

Costs were estimated from the health system perspective, considering only direct medical costs, which included diagnosis costs, treatment costs, costs attributed to the patient’s health state, and costs for the management of serious adverse events (Additional file 1: Table S4). The costs of hepatitis C virus testing were estimated based on public disclosure of medical service price items in 30 Chinese provinces and cities (22 provinces, 4 direct-administered municipalities, and 4 autonomous regions excluding Tibet), treatment costs were from Menet, and other costs were estimated from published literature [33, 44, 45]. The costs were reported in 2022 US dollars and were inflated to 2022 values using the Consumer Price Index.

Utilities were obtained from the published literature [33, 46, 47] and were made up of three components, including utility for each disease state in its natural state, utility after SVR, and utility during treatment (Additional file 1: Table S4). The utility during treatment was reduced due to the presence of adverse effects during treatment [46].

The costs and QALYs were discounted at an annual rate of 5% [48].

### Outcome measures

We report the results in terms of both effectiveness and cost-effectiveness. Effectiveness was measured by cured patient numbers, newly diagnosed cases of decompensated cirrhosis, newly diagnosed cases of hepatocellular carcinoma, need for liver transplantation, and quality-adjusted life years (QALYs). Cost-effectiveness outcomes included direct medical costs, the incremental cost-effectiveness ratio (ICER), and net health benefits. According to the recommendations of the World Health Organization and the Guidelines for the Evaluation of Chinese Pharmacoeconomics, this study used 1–3 times the per capita GDP of China reported in 2022 ($12,714–$38,142) as the willingness-to pay threshold.

### Sensitivity analyses

A one-way sensitivity analysis was performed to evaluate the impact of the uncertainty of some key model parameters (i.e. simulation of cohort characteristics parameters, costs, discount rate, SVR rates, etc.) on the results. The estimated range of each parameter was based on either the reported or determined by assuming a 5–20% change from the base-case value. We also performed a probabilistic sensitivity analysis using Monte Carlo simulations for 10,000 iterations to evaluate the uncertainty of our input parameters and gave the results by a cost-effectiveness curve.

## Results

The main results of question one are shown in Table 1. Opting for the pan-genotypic regimen resulted in an average increase of 0.13 QALYs compared to selecting the gene-specific regimen. While choosing the pan-genotypic regimen incurred an additional cost of $165, the calculated ICER was $1,268 per QALY. This meant that choosing the pan-genotypic regimen was more cost-effective, considering the ICER was less than a willingness-to-pay threshold of one time the GDP per capita. The pan-genotypic option cured 5,868 more people per 100,000 individuals, indicating superior treatment outcomes, and each additional person cured would result in an additional cost of $2,812. Furthermore, the results also showed that the use of pan-genotypic regimens would lead to an 86.5% decrease in DC cases, a 64.6% decrease in HCC cases, and a 78.2% reduction in the need for liver transplantation compared to gene-specific regimens.

**Table 1:** Model simulation results

Strategy	Cost ($)	Effectiveness (QALYs)	ICER ($/QALY)	Effectiveness (Cured)	ICER ($/Cured)	Number of new cases		
						DC	HCC	LT
Gene-specific treatment	2869	13.756		93699		2003	3874	261
Pan-genotype treatment	3034	13.886	1268	99567	2812	270	1372	57

By adjusting the simulation duration, we found that the first year in which treatment was received choosing the pan-genotypic option would incur an extra cost of $487, and resulted in an average increase of 0.001 QALYs compared to selecting the gene-specific regimen. The cost-effectiveness of choosing the pan-genotypic regimen became apparent starting from the fifth year(ICER = 32,034 $/QALY). By the tenth year, it would be less than 1 times the per capita GDP(ICER = 11,424 $/QALY). By adjusting the price of SOF/VEL, we also observed that when the unit price of SOF/VEL dropped to $427.4, pan-genotypic regimens had an absolute advantage in 30-years simulations. When the unit price of SOF/VEL decreased from $489.1 to $306.7, choosing pan-genotypic regimens became advantageous in any simulation years.

**Fig. 2:**
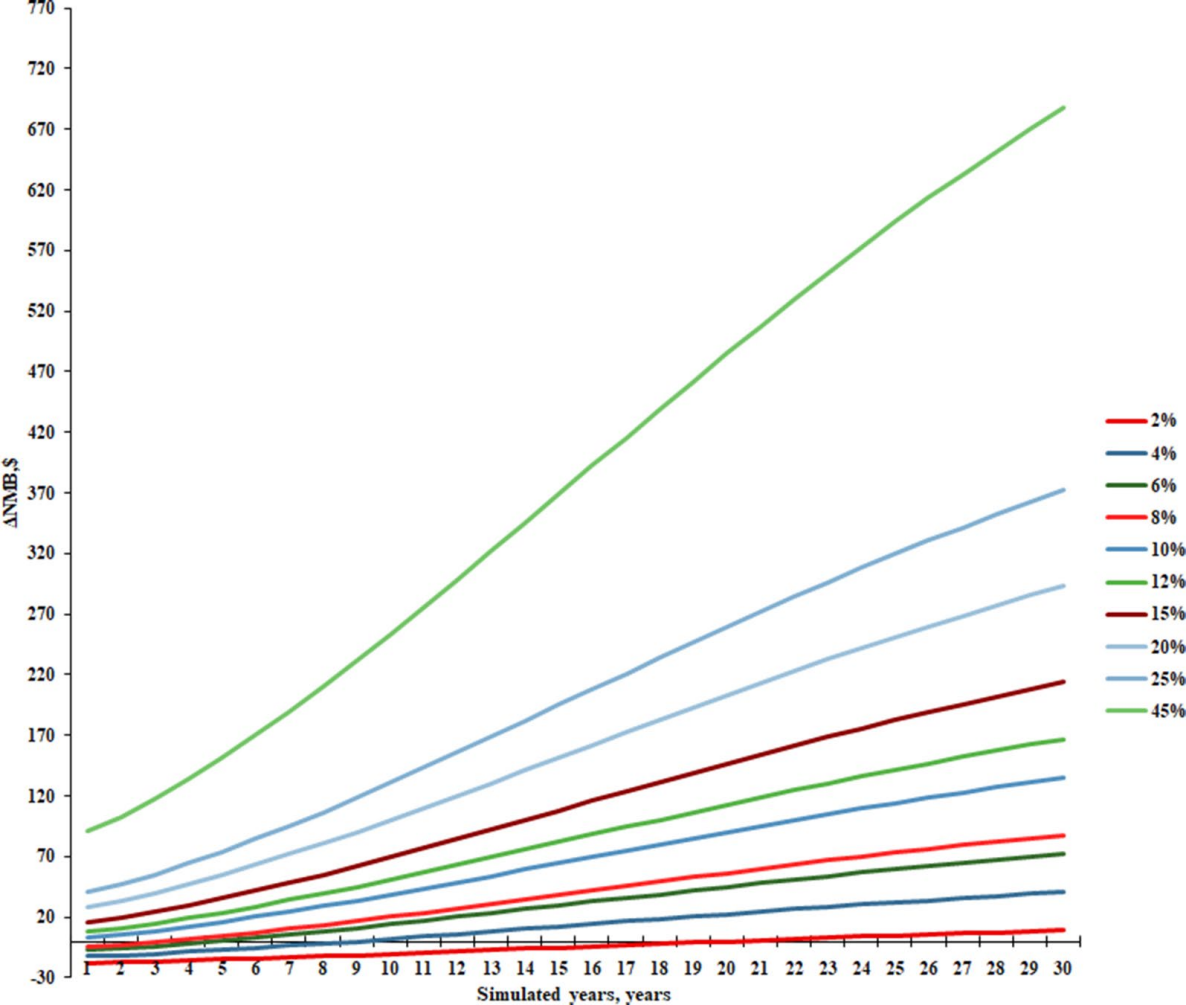
Plot of net monetary benefit margin over modeled years. *NMB* Net Monetary Benefit, *ΔNMB* NMB(identification of 3b patient group)—NMB(direct use of pan-genotype regimen

The results for the second question showed that the higher the proportion of 3b patients, the greater the cost-effectiveness, efficacy, and reduction in hepatitis C-related disease by identifying 3b patients compared to a direct pan-genotypic regimen. From a 3b patient population of 10%, the simulaiton results showed that identifying 3b patients was cost-effective and became absolutely advantageous when the proportion of 3b patients reached 12%. Moreover, as the simulation period extended, the subgroup that underwent genetic testing to identify 3b patients, in comparison to the subgroup using a pan-genotypic regimen, would incur lower costs, achieve higher average QALYs, and experience a reduced incidence of hepatitis C-related diseases. When the simulation year extended to 20 years or more, the subgroups identifying 3b patients began to exhibit advantage in all areas, except for those where the proportion of 3b patients was less than 1%. Net monetary benefits were greater than 0 for both subgroups in any simulated year and at any percentage of 3b patients. We selected ten representative proportions of 3b patients based on the proportions of 3b patients in various provinces, the change in the difference in net monetary benefits was shown in Fig. 2.

### Sensitivity analyses

**Fig. 3:**
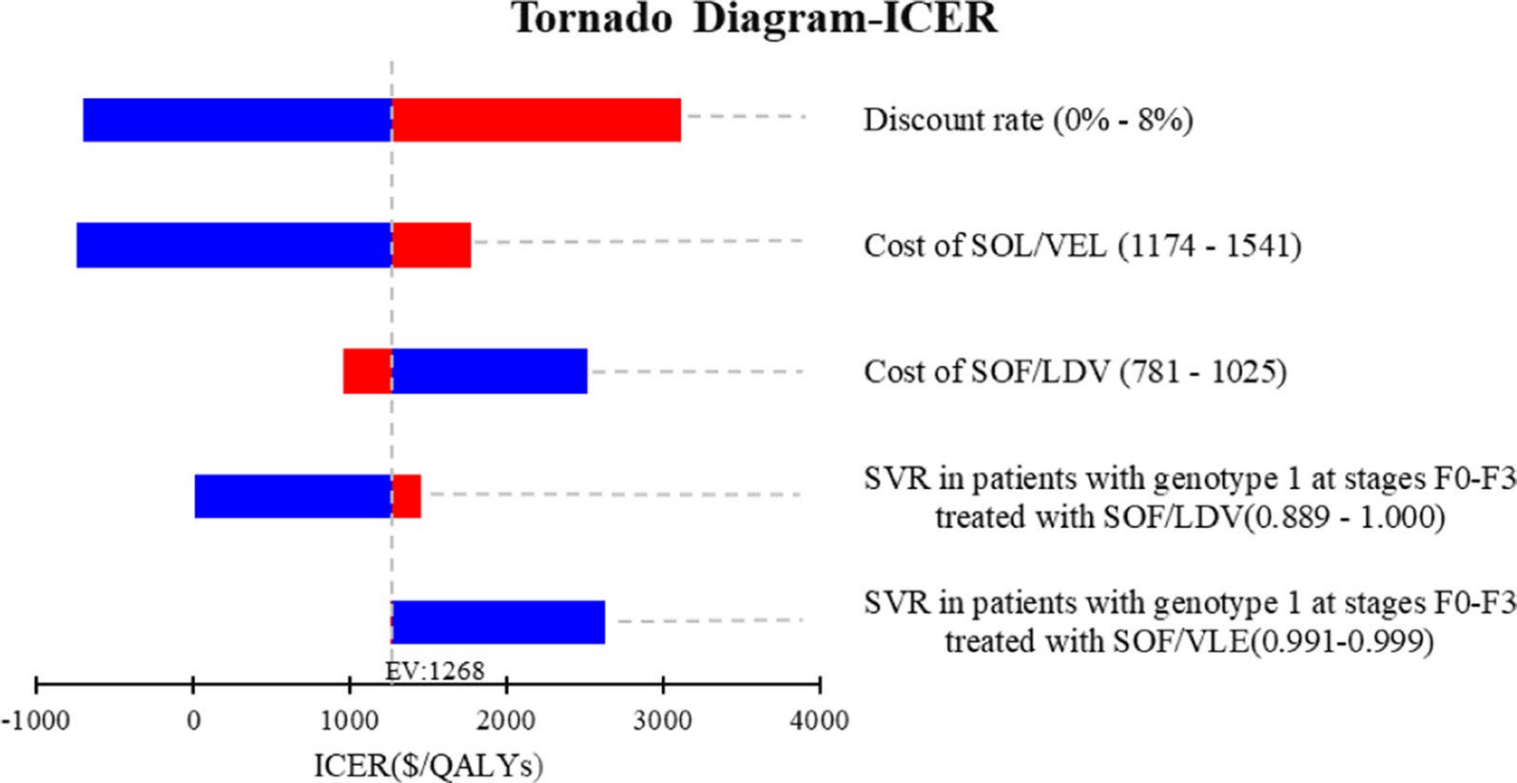
Tornado diagram of one-way sensitivity analyses. *SVR* sustained virologic response, *F0–F4* METAVIR fibrosis score, *SOF* Sofosbuvir, *VEL* Velpatasvir, *LDV* Ledipasivr

Univariate sensitivity analysis showed that the results remained essentially unchanged when the parameters fluctuated within reasonable ranges (Fig. 3). The change in parameters would only change the pan-genotypic regimen from a dominant regimen to an absolute dominant regimen.

**Fig. 4:**
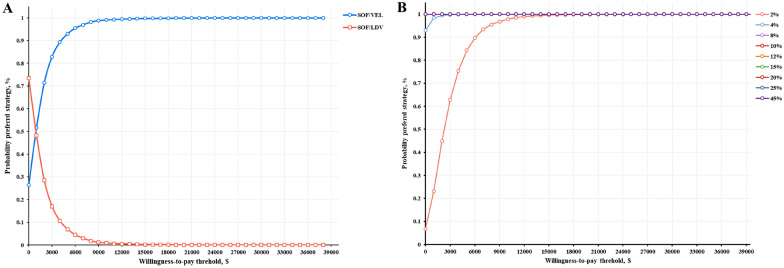
Cost-effectiveness Acceptability Curve A question 1 simulation; B question 2 simulation. *SOF* Sofosbuvir, *VEL* Velpatasvir, *LDV* Ledipasivr

The results of the probabilistic sensitivity analysis remained robust and are presented in Fig. 4. The probabilistic sensitivity analysis result of question 1 showed that gene-specific regimen was cost-effectiveness as the willingness-to-pay threshold increases from 0 to $931.1. When the willingness-to-pay exceeded $931.1, there was an increasing trend in the probability that pan-genotypic regimen was more cost-effective, ranging from 50% to 99.9%. The probabilistic sensitivity analyses for Question 2 were simulated over a 30-year period, choosing nine scenarios that represented the proportion of patients with 3b in each province and city in China. The results showed that the probability of performing genotyping tests being cost-effectiveness would be higher as the willingness-to-pay threshold increased in areas with 2% and 4% of patients with 3b, and that performing genotyping tests would always be the advantageous option when the percentage of patients with 3b was greater than 8%.

## Discussion

Our study found that pan-genotypic regimens were cost-effective in long-term simulations but may not at the begining. We find that in the short term, it is more expensive to choose the pan-genotypic regimen, but the difference in treatment costs between the two regimens becomes decreasing as the number of years of simulated treatment increases. This may be attributed to the higher drug prices for pan-genotypic regimens and the fact that choosing pan-genotypic regimens results in more patients to pay for Hepatitis C treatment, which is more expensive compared to the cost of genetic testing for the entire patient when choosing a gene-specific regimens. However, because a successful cure prevents hepatitis C from worsening, it can reduces the costs associated with hepatitis C-related illnesses. In long-term simulations, opting for pan-genotypic regimens would prove advantageous also because it enables more patients to enhance their quality of life through successful treatment. In our research, we found that the higher the number of individuals declining genotyping tests, the greater the likelihood that the utilization of pan-genotypic regimens would be cost-effective in the short term. However, it had little to no effect on long-term results. This suggests the need to consider factors beyond efficacy when selecting recommended regimens. Our study also found that in areas with a 3b prevalence of over 12%, genetic testing was still necessary before providing targeted treatment recommendations. In addition, genotype data can be obtained from sources such as local epidemiological survey databases, making this a highly feasible proposal. The direct application of our study in local practice might pose some challenges due to data availability issues, including the effectiveness of treatments for drug users, patient willingness to undergo treatment, and the availability of genetic testing equipment.

In contrast to similar studies, our long-term simulation results are consistent with the findings of Goel et al. in India [8]. They found that using velpatasvir-based pan-genotypic to treat hepatitis C increased QALYs by 0.92 and incurred an additional cost of $107, indicating cost-effectiveness. However, Gholamhoseini et al.'s [9] short-term simulations in Iran showed that the use of pan-genotypic regimens was cost-effective since they did not distinguish between differences in efficacy for genotypes 1 and 3 in the subgroups who used SOF/VEL. In comparison to previous studies [33, 39, 49], we introduced the consideration of patient attrition or discontinuation from treatment due to genetic testing and second-line treatment. We also enhanced the hepatitis C progression pathway and fine-tuned the model parameters. For instance, we incorporated the most recent prices of DAAs and utilized epidemiological data specific to China. This enabled our study to provide insights into the suitability of clinical pathways for regimen selection based on genotypic test results in the contemporary context. It also serves as an evidence base for addressing future decision-making questions related to hepatitis C management.

Our study has certain limitations. First, we did not consider the impact of disease transmission. Hepatitis C is an infectious disease, which means that uncured and untreated patients are at risk of infecting others [15]. Since hepatitis C is a curable disease, successfully curing the patient is equivalent to controlling the source of infection. Curing hepatitis C disease helps patients reintegrate into society, reducing discrimination and social exclusion caused by the illness. This contributes to the establishment of a more inclusive social environment. Recovered patients are more likely to engage in regular work and social activities, reducing productivity losses associated with the disease the burden of care on their families. The absence of this consideration in our study may lead to an underestimation of the benefits of pan-genotypic regimens. Second, due to constraints in data accessibility, we assumed consistent fibrosis severity across genotypes and did not consider the impact of treatment on patients with comorbidities, potentially differing from real clinical scenarios. Additionally, we did not address patients with compound or unknown genotypes. Actually, pan-genotypic regimens can be used to treat this group of patients, whereas gene-specific regimens cannot, which means our reasearch potentially underestimating the cost-effectiveness of pan-genotypic regimens. To overcome these limitations, further research, clinical trials, and better data are needed. High-quality methodology in infectious disease cost-effectiveness evaluation can help address these issues. As an infectious disease in the process of being eliminated, the recording and analysis of data related to the transmission of hepatitis C will help in the prevention and control of other infectious diseases.

## Conclusion

Choosing pan-genotypic regimens can be cost-effective by preventing disease progression. We recommend prioritizing pan-genotypic regimens for overall population health. In regions with 12% or more genotype 3b patients, genotyping tests should still be conducted even when promoting pan-genotypic regimens.

### Supplementary Information

**Additional file 1: Table S1.** SVR rate and incidence of serious adverse reactions for different treatment regimens. **Table S2.** SVR rate and incidence of serious adverse reactions for different treatment regimens. **Table S3.** Annual transition probabilities used in the markov model. **Table S4.** Cost and utility parameters used in the markov model.

## Data Availability

All data and material are available from the corresponding author upon reasonable request.

## Abbreviations

DAAs: Direct antiviral agents
SOF/LDV: Sofosbuvir/ledipasvir
SOF/VEL: Sofosbuvir/velpatasvir
GZR/EBR: Grazoprevir/elbasvir
SOF/VEL/VOX: Sofosbuvir/velpatasvir/voxilaprevir
RBV: Ribavirin
SVR: Sustained virologic response
QALY: Quality-adjusted life years
ICER: Incremental cost-effectiveness ratio
GDP: Gross domestic product
CHEERS: Consolidated Health Economic Evaluation Reporting Standards
DC: Decompensated cirrhosis
HCC: Hepatocellular carcinoma
