# Supplementary material for: Is it time for China to prioritize pan-genotypic regimens for treating patients with hepatitis C?
Source: Cost Eff Resour Alloc. 2024 Feb 6;22:11. doi: 10.1186/s12962-024-00519-2 (PMC10848349; doi:10.1186/s12962-024-00519-2)
Supplement: Supplementary file 1 — Additional file 1: Table S1. SVR rate and incidence of serious adverse reactions for different treatment regimens. Table S2. SVR rate and incidence of serious adverse reactions for different treatment regimens. Table S3. Annual transition probabilities used in the markov model. Table S4. Cost and utility parameters used in the markov model. [file 12962_2024_519_MOESM1_ESM.docx]

Additional file1

**TABLE S1** SVR Rate and Incidence of Serious Adverse Reactions for Different Treatment Regimens

**TABLE S2** SVR Rate and Incidence of Serious Adverse Reactions for Different Treatment Regimens

**TABLE S3** Annual Transition Probabilities Used in the Markov model

**TABLE S4** Cost and Utility Parameters Used in the Markov Model

SUPPLEMENTARY REFERENCES

**TABLE S1** SVR rate and incidence of serious adverse reactions for different treatment regimens

| **Model Parameter** | | **Base case** | **Range** | **Reference** |
| --- | --- | --- | --- | --- |
| **SVR Rates（SOF/VEL for 12 weeks）** | | | | |
| F0-3 | GT-1 | 99.7% | 0.991 – 0.999 | ^1^ |
|  | GT-2 | 99.3% | 0.984 – 0.997 | ^1^ |
|  | GT-3a | 93.5% | 0.786 – 0.992 | ^2^ |
|  | GT-3b | 89.3% | 0.718 - 0.977 | ^2^ |
|  | GT-6 | 97.7% | 0.934 – 1.000 | ^1^ |
| F4 | GT-1 | 100.0% | 0.692 – 1.000 | ^2^ |
|  | GT-2 | 100.0% | 0.715 – 1.000 | ^2^ |
|  | GT-3a | 100.0% | 0.715 – 1.000 | ^2^ |
|  | GT-3b | 50.0% | 0.230 – 0.770 | ^2^ |
|  | GT-6 | 100.0% | 0.735 – 1.000 | ^2^ |
| **SVR Rates（SOF/LDV for 12 weeks）** | | | | |
| F0-3 | GT-1 | 98.8% | 0.889 - 1.000 | ^3^ |
|  | GT-2 | 98.4% | 0.885 - 1.000 | ^3^ |
|  | GT-6 | 98.6% | 0.887 - 1.000 | ^3^ |
| F4 | GT-1 | 99.0% | 0.891 - 1.000 | ^3^ |
|  | GT-2 | 97.5% | 0.878 - 1.000 | ^3^ |
|  | GT-6 | 98.1% | 0.883 - 1.000 | ^3^ |
| **SVR Rates（SOF/VEL/VOX for 12 weeks）** | | | | |
| F0-4 | GT-1 | 97.4% | 0.828 - 1.000 | ^4^ |
|  | GT-2 | 94.9% | 0.873 - 1.000 | ^4^ |
|  | GT-3 | 86.7% | 0.783 – 0.957 | ^4^ |
|  | GT-6 | 100.0% | 0.900 - 1.000 | ^4^ |
| **SVR Rates（SOF/VEL + RBV for 12 weeks）** | | | | |
| F0-3 | GT-3b | 94.2% | 0.820 – 0.970 | ^5^ |
| F4 | GT-3b | 81.3% | 0.820 – 0.970 | ^5^ |
| **Serious AE Rates** | | | | |
| SOF/VEL | | 2.3% | 2.1% - 2.5% | ^1^ |
| SOF/VEL + RBV | | 1.5% | 1.3% - 1.6% | ^5^ |
| SOF/LDV | | 1.0% | 0.9% - 1.1% | ^3^ |
| SOF/VEL/VOX | | 2.0% | 1.8% - 2.2% | ^6^ |

*SVR*, sustained virologic response; *AE*, adverse events; *SOF*, Sofosbuvir; *VEL*, Velpatasvir; *LDV*, Ledipasivr; *VOX*, Voxilaprevir; *RBV*, ribavirin; *F0–F4*, METAVIR fibrosis score; *GT*, genotype.

**TABLE S2** Annual transition probabilities used in the markov model

| **Transition probabilities** | **Base case** | **Range** | **Reference** |
| --- | --- | --- | --- |
| **Nature Progression** | | | |
| F0→F1 | 0.117 | 0.104 - 0.130 | ^7^ |
| F1→F2 | 0.085 | 0.075 - 0.096 | ^7^ |
| F2→F3 | 0.120 | 0.109 - 0.133 | ^7^ |
| F3→F4 | 0.116 | 0.104 - 0.129 | ^7^ |
| F3→DC | 0.012 | 0.010 - 0.014 | ^8^ |
| F3→HCC | 0.011 | 0.009 - 0.013 | ^8^ |
| F4→DC | 0.039 | 0.010 - 0.079 | ^9^ |
| F4→HCC | 0.014 | 0.010 - 0.079 | ^10^ |
| DC→HCC | 0.068 | 0.030 - 0.083 | ^10^ |
| DC→LT | 0.023 | 0.010 - 0.062 | ^10^ |
| DC→Death | 0.104 | 0.069 - 0.052 | ^11^ |
| HCC→LT | 0.040 | 0.000 - 0.140 | ^10^ |
| HCC→Death | 0.427 | 0.330 - 0.860 | ^10^ |
| LT→Death | 0.116 | 0.060 - 0.420 | ^10^ |
| PLT→Death | 0.044 | 0.024 - 0.110 | ^10^ |
| **Disease Progression after SVR** | | | |
| F3 SVR→F2 SVR | 0.500 | 0.250 - 0.820 | ^8^ |
| F4 SVR→F2 SVR | 0.080 | 0.060 - 0.010 | ^8^ |
| F4 SVR→F3 SVR | 0.340 | 0.190 - 0.490 | ^8^ |
| F3 SVR→DC | 0.001 | 0.0008 - 0.0012 | ^8^ |
| F3 SVR→HCC | 0.007 | 0.006 - 0.008 | ^8^ |
| F4 SVR→DC | 0.003 | 0.002 - 0.004 | ^8^ |
| F4 SVR→HCC | 0.006 | 0.005 - 0.008 | ^9^ |

*SVR*, sustained virologic response; *F0–F4*, METAVIR fibrosis score; *DC*, decompensated cirrhosis; *HCC*, hepatocellular carcinoma; *LT*, liver transplantation (first year); *PLT*, post liver transplantation (> 1 year).

**TABLE S3** Population characteristics of HCV-Infected patients in China

| **Variable** | | **Base case** | **Range** | **Reference** |
| --- | --- | --- | --- | --- |
| **Age Distribution of HCV-infected Population** | | | | ^12^ |
|  | Genotype 1 | 47 | 39 – 57 |  |
|  | Genotype 2 | 48 | 39 – 58 |  |
|  | Genotype 3 | 38 | 32 – 42 |  |
|  | Genotype 6 | 35 | 31 – 41 |  |
| **Fibrosis Distribution of HCV-infected Population** | | | | ^13^ |
|  | F0 | 17.0% | 13.0% - 21.0% |  |
|  | F1 | 35.0% | 20.0% - 51.0% |  |
|  | F2 | 22.0% | 17.0% - 27.0% |  |
|  | F3 | 14.0% | 10.0% - 17.0% |  |
|  | F4 | 12.0% | 9.0% - 15.0% |  |
| Proportion of Patients Receiving Pan-genotypic Therapy Requiring Genotype Testing | | 35.2% | 33.5% - 37.0% | ^14^ |
| **Proportion of patients by genotype nationwide** | | | | ^14^ |
|  | Genotype 1 | 52.4% | 50% - 54.7% |  |
|  | Genotype 2 | 28.8% | 27.3% - 30.2% |  |
|  | Genotype 3a | 4.6% | 4.4% - 4.8% |  |
|  | Genotype 3b | 7.0% | 6.6% - 7.3% |  |
|  | Genotype 6 | 7.3% | 6.9% - 7.6% |  |
| **Proportion of patients by genotype at 3b<5% regions** | | | | ^14^ |
|  | Genotype 1 | 54.5% | 52.2%- 56.8% |  |
|  | Genotype 2 | 40.9% | 38.9% - 43.0% |  |
|  | Genotype 3a | 2.0% | 1.9% - 2.1% |  |
|  | Genotype 3b | 1.6% | 1.5% - 1.7% |  |
|  | Genotype 6 | 0.9% | 0.9% - 1.0% |  |
| **Proportion of patients by genotype at 3b>5% regions** | | | | ^14^ |
|  | Genotype 1 | 48.5% | 45.9% - 51.0% |  |
|  | Genotype 2 | 6.4% | 6.1% - 6.7% |  |
|  | Genotype 3a | 9.4% | 8.9% - 9.8% |  |
|  | Genotype 3b | 16.9% | 16.0% - 17.7% |  |
|  | Genotype 6 | 18.9% | 18.0% - 19.9% |  |

*F0*, no fibrosis; *F1*, portal fibrosis without septa; *F2*, portal fibrosis with few septa; *F3*, numerous septa without cirrhosis; *F4*, cirrhosis.

**TABLE S4** Cost and utility parameters used in the markov model

| **Model Parameter** | **Base case** | **Range** | **Reference** |
| --- | --- | --- | --- |
| **Testing Cost(one - time)** | | | |
| Anti-HCV detection($) | 6.0 | 4.8 – 7.2 | Caculated |
| HCV-RNA detection($) | 10.7 | 8.6 – 12.9 | Caculated |
| genotyping test($) | 23.9 | 19.1 – 28.7 | Caculated |
| **Treatment cost (one course of treament)** | | | |
| SOF/VEL($) | 1467 | 1174 - 1541 | ^15^ |
| SOF/LDV($) | 976 | 781 – 1025 | ^15^ |
| SOF/VEL/VOX($) | 3235 | 2588 – 3396 | ^15^ |
| RBV($) | 5 | 3.87 – 5.80 | ^15^ |
| **Health state costs (annual)** | | | |
| F0-3 | 130 | 104 – 156 | ^16^ |
| F4 | 801 | 641 – 961 | ^16^ |
| DC | 1407 | 1125 - 1688 | ^16^ |
| HCC | 3895 | 3116 - 4674 | ^16^ |
| LT | 59222 | 42790 - 85580 | ^17^ |
| PLT | 9414 | 8558 - 10522 | ^17^ |
| **Others** |  |  |  |
| RR of cost post SVR | 0.709 | 0.592 – 0.855 | ^17^ |
| serious AE management costs | 1182 | 946 - 1418 | ^18^ |
| **Utility of disease states** |  |  |  |
| F0 | 0.878 | 0.751 – 0.985 | ^19^ |
| F1 | 0.878 | 0.751 – 0.985 | ^19^ |
| F2 | 0.863 | 0.701 – 0.985 | ^19^ |
| F3 | 0.863 | 0.701 – 0.985 | ^19^ |
| F4 | 0.792 | 0.670 – 0.907 | ^19^ |
| DC | 0.690 | 0.570 – 0.950 | ^17^ |
| HCC | 0.685 | 0.532 – 0.821 | ^19^ |
| LT | 0.663 | 0.563 – 0.800 | ^19^ |
| PLT | 0.773 | 0.636 – 0.850 | ^19^ |
| **Utility after achieving SVR** |  |  |  |
| F0 SVR | 0.928 | 0.806 – 1.000 | ^19^ |
| F1 SVR | 0.928 | 0.806 – 1.000 | ^19^ |
| F2 SVR | 0.911 | 0.791 – 1.000 | ^19^ |
| F3 SVR | 0.893 | 0.766 – 1.000 | ^19^ |
| F4 SVR | 0.850 | 0.722 – 0.955 | ^19^ |
| **Utility during treatment** |  |  |  |
| utility reduction value  (Achieve SVR) | -0.03 | -0.032 - -0.029 | ^20^ |
| utility reduction value  (Treatment failure) | -0.05 | -0.053 - -0.048 | ^20^ |

*SOF*, Sofosbuvir; *VEL*, Velpatasvir; *LDV*, Ledipasivr; *VO*X, Voxilaprevir; *RBV*, ribavirin; *SVR*, sustained virologic response; *AE*, adverse events; *F0–F4*, METAVIR fibrosis score; *DC*, decompensated cirrhosis; *HCC*, hepatocellular carcinoma; *LT*, liver transplantation (first year); PLT = post liver transplantation (> 1 year).

**References:**

1. Liu CH, Chen PY, Chen JJ et al. Sofosbuvir/velpatasvir for patients with chronic hepatitis C virus infection and compensated liver disease: real-world data in Taiwan. *Hepatol Int*. 2021;15(2):338-349

2. Wei L, Lim SG, Xie Q et al. Sofosbuvir-velpatasvir for treatment of chronic hepatitis C virus infection in Asia: a single-arm, open-label, phase 3 trial. *Lancet Gastroenterol Hepatol*. 2019;4(2):127-134

3. Lo CC, Huang CF, Cheng PN et al. Ledipasvir/sofosbuvir for HCV genotype 1, 2, 4-6 infection: Real-world evidence from a nationwide registry in Taiwan. *J Formos Med Assoc*. 2022;121(8):1567-1578

4. Xie J, Xu B, Wei L, Huang C, Liu W. Effectiveness and Safety of Sofosbuvir/Velpatasvir/Voxilaprevir as a Hepatitis C Virus Infection Salvage Therapy in the Real World: A Systematic Review and Meta-analysis. *Infect Dis Ther*. 2022;11(4):1661-1682

5. Huang R, Rao H, Xie Q et al. Comparison of the efficacy of sofosbuvir plus ribavirin in Chinese patients with genotype 3a or 3b HCV infection. *J Med Virol*. 2019;91(7):1313-1318

6. Liu CH, Peng CY, Liu CJ et al. Sofosbuvir/velpatasvir/voxilaprevir for patients with chronic hepatitis C virus infection previously treated with NS5A direct-acting antivirals: a real-world multicenter cohort in Taiwan. *Hepatol Int*. 2023;

7. Thein HH, Yi Q, Dore GJ, Krahn MD. Estimation of stage-specific fibrosis progression rates in chronic hepatitis C virus infection: a meta-analysis and meta-regression. *Hepatology*. 2008;48(2):418-31

8. Hagan LM, Sulkowski MS, Schinazi RF. Cost analysis of sofosbuvir/ribavirin versus sofosbuvir/simeprevir for genotype 1 hepatitis C virus in interferon-ineligible/intolerant individuals. *Hepatology*. 2014;60(1):37-45

9. Dienstag JL, Ghany MG, Morgan TR et al. A prospective study of the rate of progression in compensated, histologically advanced chronic hepatitis C. *Hepatology*. 2011;54(2):396-405

10. Wu J, Zhou Y, Fu X et al. The Burden of Chronic Hepatitis C in China From 2004 to 2050: An Individual-Based Modeling Study. *Hepatology*. 2019;69(4):1442-1452

11. Wang SB, Wang JH, Chen J, Giri RK, Chen MH. Natural history of liver cirrhosis in south China based on a large cohort study in one center: a follow-up study for up to 5 years in 920 patients. *Chin Med J (Engl)*. 2012;125(12):2157-62

12. Rao H, Wei L, Lopez-Talavera JC et al. Distribution and clinical correlates of viral and host genotypes in Chinese patients with chronic hepatitis C virus infection. *J Gastroenterol Hepatol*. 2014;29(3):545-53

13. Chen GF, Wei L, Chen J et al. Will Sofosbuvir/Ledipasvir (Harvoni) Be Cost-Effective and Affordable for Chinese Patients Infected with Hepatitis C Virus? An Economic Analysis Using Real-World Data. *PLoS One*. 2016;11(6):e0155934

14. Chen Y, Yu C, Yin X et al. Hepatitis C virus genotypes and subtypes circulating in Mainland China. *Emerg Microbes Infect*. 2017;6(11):e95

15. MENET - China Pharmaceutical Information Database. 2023;2023(2023-4-16)

16. Heffernan A, Ma Y, Nayagam S et al. Economic and epidemiological evaluation of interventions to reduce the burden of hepatitis C in Yunnan province, China. *PLoS One*. 2021;16(1):e0245288

17. Wei X, Zhao J, Yang L. Cost-effectiveness of new antiviral treatments for non-genotype 1 hepatitis C virus infection in China: a societal perspective. *BMJ Glob Health*. 2020;5(11)

18. Qing-ping S, Xiao-dong J, Feng D et al. Consequences, measurement, and evaluation of the costs associated with adverse drug reactions among hospitalized patients in China. *BMC Health Serv Res*. 2014;14:73

19. Zhou HJ, Cao J, Shi H et al. Cost-Effectiveness Analysis of Pan-Genotypic Sofosbuvir-Based Regimens for Treatment of Chronic Hepatitis C Genotype 1 Infection in China. *Front Public Health*. 2021;9:779215

20. Younossi ZM, Singer ME, Mir HM, Henry L, Hunt S. Impact of interferon free regimens on clinical and cost outcomes for chronic hepatitis C genotype 1 patients. *J Hepatol*. 2014;60(3):530-7
